# Tracing the patterns of HIV-1 transmission among people with different diagnosis status in Greece

**DOI:** 10.1016/j.virusres.2026.199803

**Published:** 2026-09-16

**Authors:** Evangelia Georgia Kostaki, Stefanos Limnaios, Georgios Adamis, Georgios Xylomenos, Maria Chini, Nikos Mangafas, Marios Lazanas, Stavros Patrinos, Simeon Metallidis, Olga Tsachouridou, Vasileios Papastamopoulos, Dimitrios Chatzidimitriou, Anastasia Antoniadou, Antonios Papadopoulos, Konstantinos Protopapas, Chrysa Tsiara, Mina Psichogiou, Dimitrios Basoulis, Dimitrios Pilalas, Dimitra Paraskeva, Georgios Chrysos, Vasileios Paparizos, Sofia Kourkounti, Helen Sambatakou, Vasileios Bolanos, Nikolaos V. Sipsas, Malvina Lada, Emmanouil Barbounakis, Evrikleia Kantzilaki, Periklis Panagopoulos, Vasilis Petrakis, Stelios Drimis, Pagona Lagiou, Angelos Hatzakis, Lemonia Skoura, Gkikas Magiorkinis, Dimitrios Paraskevis

**Affiliations:** a Department of Preventive Medicine, Epidemiology and Medical Statistics, Medical School, National and Kapodistrian University of Athens, Athens, Greece; b 1st Department of Internal Medicine, G. Genimatas General Hospital, Athens, Greece; c 3rd Department of Internal Medicine-Infectious Diseases Unit, "Korgialeneio-Benakeio" Red Cross General Hospital, Athens, Greece; d National Public Health Organization, Marousi, Greece; e 1st Department of Internal Medicine, AHEPA University Hospital, Medical School, Aristotle University of Thessaloniki, Thessaloniki, Greece; f 5th Department of Internal Medicine and Infectious Diseases, Evaggelismos General Hospital, Athens, Greece; g National AIDS Reference Centre of Northern Greece, Department of Microbiology, Medical School, Aristotle University of Thessaloniki, Thessaloniki, Greece; h 4th Department of Internal Medicine, Αttikon University General Hospital, Medical School, National and Kapodistrian University of Athens, Athens, Greece; i 1st Department of Internal Medicine, Laiko General Hospital, Medical School, National and Kapodistrian University of Athens, Athens, Greece; j Department of Internal Medicine, Tzaneio General Hospital, Piraeus, Greece; k HIV/AIDS Unit, A. Syngros Hospital of Dermatology and Venereology, Athens, Greece; l HIV Unit, 2nd Department of Internal Medicine, Hippokration University General Hospital, Medical School, National and Kapodistrian University of Athens, Athens, Greece; m Department of Pathophysiology, Laiko General Hospital, Medical School, National and Kapodistrian University of Athens, Athens, Greece; n 2nd Department of Internal Medicine, Sismanogleio General Hospital, Athens, Greece; o Department of Internal Medicine, University Hospital of Heraklion "PAGNI", Medical School, University of Crete, Heraklion, Greece; p 2nd Department of Internal Medicine, University General Hospital of Alexandroupolis, Democritus University of Thrace, Alexandroupolis, Greece

**Keywords:** HIV, Late presentation, Diagnosis status, Infection date, Molecular epidemiology, Phylogeography, Greece

## Abstract

Late presentation remains a major hurdle for HIV management and prevention. We investigated the potential source of transmission among people living with HIV (PLHIV) with different stages of diagnosis, using a combined approach of the traditional definition of late presentation (CD4-based) and the estimated time between HIV acquisition and diagnosis. The study included 3,093 HIV-1 subtype A1 and B sequences from a period with dense sampling in Greece. HIV acquisition dates were estimated using molecular clock analysis and transmissions patterns were assessed with a modified phylogeographic approach. For subtype A1, analysis using the estimated time from HIV acquisition to diagnosis revealed that among PLHIV with recent diagnosis, the mean number of infections originating from PLHIV with late and intermediate diagnosis, were 35.7 (11%) and 32.8 (10.2%), respectively. For those diagnosed late, 21.3 (6.7%) and 18.5 (5.8%) infections (mean number) were from PLHIV with intermediate and recent diagnosis, respectively. Similarly for subtype B, a considerable proportion of infections among PLHIV with recent (12.2%) and intermediate (13.1%) diagnoses originated from PLHIV with late status. Statistical phylogeography confirmed that late presenters were a significant source of transmission to PLHIV in other diagnosis-status categories for both subtypes. Analysis using CD4 cell count showed that late presentation and advanced disease groups provided a significant source of transmission to other groups. Our study highlights that early diagnosis, besides the beneficial effect for the disease prognosis, would significantly mitigate onward HIV transmission. This underscores the need to enhance HIV testing strategies in Greece to ensure earlier diagnosis and timely treatment initiation.

## Highlights

• Late HIV presenters are a major source of onward transmission in Greece.
• Recently diagnosed cases seeded few onward transmissions despite being the largest group.
• Consistent findings across subtypes A1 and B and two late-presentation definitions.
• Modified statistical phylogeography traced HIV transmission sources.
• Earlier HIV testing and diagnosis is of major importance to interrupt transmission.

## 1 Introduction

HIV remains a persistent global public health problem (https://www.who.int/). According to the World Health Organization (WHO), 40.8 million people were living with HIV (PLHIV) and 630,000 people died of AIDS-related illnesses in 2024 (http://www.UNAIDS.org). Although there is currently an increased focus on testing and linkage to care, many people throughout the globe are still diagnosed late. Late presentation of HIV infection is defined as diagnosis with a CD4 count below 350 cells/μL or with an AIDS-defining event, regardless of the CD4 cell count (Antinori et al, 2011). In the WHO European Region, 54.2% of people presented late in 2024 (ECDC/WHO, 2025). In the European Union/European Economic Area (EU/EEA), the proportion of people diagnosed late was higher among heterosexuals and people who inject drugs (PWID), and it also increased with age (ECDC/WHO, 2025). Although there is a declining trend in the proportion of late diagnoses in EU/EEA over time (from 51.4% in 2013 to 48.0% in 2024), late presentation remains a major hurdle for the management and prevention of HIV infection (ECDC/WHO, 2025).

In Greece, late presentation for HIV is a major health issue as in most countries in the EU/EEA. In 2024, the proportion of those diagnosed late in Greece was 53.7% (NPHO, 2025). In the context of a previous study, we used a nationwide sample of PLHIV whose sequences were found within local molecular transmission clusters (MTCs) of subtypes A1 and B (predominant HIV-1 clades), to investigate the characteristics of late presentation in Greece (Kostaki et al, 2022b). By using both the traditional definition of late presentation that is based on the CD4 cell count or AIDS-defining condition at diagnosis and the estimated time between HIV infection and diagnosis, we found that older age, heterosexual mode of HIV acquisition, and more recent diagnosis were associated with increased risk for late presentation (Kostaki et al, 2022b). Also, by using the estimated HIV infection dates - an unbiased marker for late presentation - we showed that the time to diagnosis was increasing gradually with age (Kostaki et al, 2022b).

In the current study, we aimed to investigate the potential source of HIV transmission among PLHIV with different stages of diagnosis using molecular epidemiology methods, including modified phylogeographic analysis, and a combined approach of the traditional definition of late presentation (CD4 cell count/AIDS-defining condition at diagnosis) and the estimated time since infection (time interval between HIV acquisition and diagnosis dates).

## 2 Materials and methods

### 2.1 Study sample

Our study dataset consisted of 3,093 HIV-1 sequences sampled during 1999-2015 across Greece [Central/Southern Greece (n=2,351; 76%), Northern Greece (n=694; 22.4%), Crete (n=48; 1.6%)]. Sequences were available in the protease and partial reverse transcriptase (PR/RT) regions (*pol* gene) with a length of 897 nucleotides (nt) after editing. The HXB2 positions of the HIV-1 fragment analysed were 2262-3286. We defined late presentation according to the European late presenter consensus definition proposed by Antinori et al. (2011). Data on CD4 cell count and AIDS-defining condition at diagnosis were obtained from the National HIV-1 surveillance system (https://www.eody.gov.gr), as well as the date of the first sample obtained for HIV-RNA or genotypic resistance testing. Regarding the AIDS-defining condition, the relevant information was based on the CDC-defined AIDS-defining clinical conditions. All experiments were performed in compliance with relevant guidelines and regulations. For this study, fully anonymized nucleotide sequences were retrospectively analyzed; thus, no written informed consent was required.

The Ethics Committee of the Medical School of the National and Kapodistrian University of Athens had approved this study (1516010195-08/12/2015).

### 2.2 HIV-1 subtyping, phylogenetic analysis, genetic distances estimation and molecular clock analysis

The whole procedure of HIV-1 subtyping, investigation of HIV-1 dispersal patterns and MTCs identification through phylogenetic analysis, as well as estimation of HIV infection dates by molecular clock analysis have been described in detail elsewhere (Kostaki et al, 2020; Limnaios and Kostaki et al, 2022). In brief, COMET and REGA online subtyping tools and phylogenetic analysis were used to determine the HIV-1 subtypes of the study sequences (Kostaki et al, 2020). Phylogenetic analysis based on the maximum likelihood (ML) method was performed on 1,751 subtype A1 and 2,575 subtype B sequences sampled in Greece along with 1,500 subtype A1 and 2,000 subtype B global reference sequences, to investigate the dispersal patterns of these clades and to identify MTCs (Kostaki et al, 2020). The following criteria were set for the definition of MTCs: (i) phylogenetic clusters including at least two sequences from Greece, accounting for >70% of all sequences within the cluster (geographic criterion), and (ii) large MTCs including >10 sequences with a ML transfer bootstrap expectation (TBE) >75% or posterior probability >0.85 (phylogenetic confidence criterion) (Kostaki et al, 2020). For the estimation of HIV infection dates, molecular clock analysis based on the Bayesian birth-death models was performed on 3,093 sequences (1,533 of subtype A1 and 1,560 of subtype B) found within MTCs consisting of more than 10 sequences (large clusters) (Limnaios and Kostaki et al, 2022). Regarding the inclusion of people on ART, our previous studies have shown that molecular clock analysis provides reasonable estimates of the time to the most recent common ancestor (t_MRCA_) and narrow highest posterior density (HPD) intervals when the proportion of people on ART in the analysis is below 30% (Limnaios and Kostaki et al, 2022). In the present study, this proportion was substantially lower, at 7.7%, and would reach a maximum of 11.4% if all individuals with an unknown treatment status were considered to be receiving treatment. Therefore, we decided to retain these individuals in the analysis, both to increase the sample size and, more importantly, to improve sampling coverage.

The pairwise genetic distances were estimated for sequences found within the large MTCs of subtypes A1 and B. The analysis was performed separately for each subtype sequences (A: 1,533, B: 1,560) using the Tamura-Nei nucleotide substitution model in MEGA X (Kumar et al, 2018).

### 2.3 Modified phylogeographic analysis

Modified phylogeographic analysis was conducted on a subset of the study data consisting of 2,385 (subtype A1: 1,229, subtype B: 1,156) sequences previously found within large MTCs with an estimated pairwise genetic distance ≤ 0.015 substitutions per site.

In brief, the modified phylogeographic analysis was performed separately for the two approaches used to define diagnosis status, using the corresponding sequence datasets after the respective exclusions. Thus, each approach was analysed using a separate set of bootstrap-reconstructed ML phylogenetic trees. Within each analysis, the diagnosis-status category of each sequence was assigned as a character state to the corresponding tip of each bootstrap tree. HIV-1 migration events between diagnosis-status categories were then inferred by maximum parsimony and compared with those obtained after random reshuffling of the diagnosis-status assignments among the tips to assess whether the observed transmission patterns differed from those expected by chance.

Sequences of each subtype were analysed separately to estimate the number of viral migration events (i.e., HIV transmissions) occurring between PLHIV with different diagnosis-status categories. For each approach, sequences were assigned to diagnosis-status categories according to the respective definition. Diagnosis status was defined either by the traditional definition of late presentation or the estimated time from infection to diagnosis. Based on the traditional definition of late presentation, sequences were grouped into four categories according to the CD4 cell count or presence of an AIDS-defining condition at diagnosis. Specifically, according to this approach, a diagnosis was characterized as “advanced disease” when a person’s CD4 count at diagnosis was < 200 cells/μL or the person was presented with an AIDS-defining condition, as “late” when a person was diagnosed with 200 ≤ CD4 count ≤ 350 cells/μL, as “non-late” when the person’s CD4 count was > 350 cells/μL, and as “unknown” when the presentation status of the person was not known (Antinori et al, 2011). From this analysis, we excluded the sequences found within three MTCs of PWID; two MTCs for subtype A1 (n=79 sequences), and one for subtype B (n=112 sequences). The total number of sequences assigned to diagnosis-status categories according to the traditional definition of late presentation was 1,150 for subtype A1 and 1,044 for subtype B.

Using the alternative approach based on the estimated time interval between HIV acquisition and diagnosis dates, four diagnosis-status categories were considered. Sequences from treatment-naïve PLHIV were categorized into tertiles and diagnosis was characterized as “recent” (in years, A1: 0.05-1.31, B: 0.09-1.09), “intermediate” (in years, A1: 1.32-2.68, B: 1.10-2.41), and “late” (in years, A1: 2.69-11.54, B: 2.42-11.87). Sequences from treated PLHIV or people with an unknown treatment status formed a separate group. The median estimate of the t_MRCA_ of a clade of taxa on a dated tree, as estimated by the molecular clock analysis, was used to approximate the HIV acquisition date, and the date of the first sample obtained for HIV-RNA or genotypic resistance testing was used to approximate the HIV diagnosis date of a person (Kostaki et al, 2021; Kostaki et al, 2024). From this analysis, we also excluded the sequences found within the three PWID MTCs [two MTCs for subtype A1 (n=79) and one for subtype B (n=112)], and a number of sequences from treatment-naïve PLHIV for whom the estimated time from infection to diagnosis was either negative or >12 years (74 for subtype A1 and 70 for subtype B). The total number of sequences assigned to diagnosis-status categories based on the alternative definition of late presentation was 1,076 for subtype A1 and 974 for subtype B.

HIV-1 migration events between the different diagnosis-status categories of each approach were inferred for each bootstrap tree (as generated by the ML method under the GTR+Γ substitution model in RAxML v8.2.10 (Stamatakis, 2014)) by using the maximum parsimony method as implemented in PAUP*4.0 (Swofford, 2002). This method has been applied in several studies and has been described in detail elsewhere (Angelis et al, 2015; Magiorkinis et al, 2016; Kostaki et al, 2018; Kostaki et al, 2022a). In brief, each category was represented by a character which was assigned at the tips of the 350 bootstrap-reconstructed ML phylogenetic trees of each approach and subtype, and then the ancestral character states were reconstructed at the trees’ nodes by parsimony. By estimating the total number of character changes over all the bootstrap trees, we obtained an estimate of the viral migration events. Subsequently, to determine whether the inferred migration events were significantly different from those expected to occur by chance, we used a modified statistical phylogeographic approach. In the context of this analysis, we re-estimated the migration events over the bootstrap trees after a random reshuffling of the diagnosis-status assignments at the tips performed on Mesquite v.3.521 (Maddison WP and Maddison DR, 2019), and then we compared the distribution of events estimated over these trees with the distribution of events estimated over the original trees using the one-sided Mann-Whitney U test. We assumed that random reshuffling of the diagnosis-status assignments at a tree’s tips represents the null hypothesis of random mixing of diagnosis-status categories, under which HIV transmission between individuals belonging to different diagnosis-status categories is equally likely. The significance level for the distributions’ comparison was set at 0.05, and an adjustment was made to p-values (Bonferroni correction) to counteract the multiple comparisons problem. As a result, in our study, for both subtypes, statistically significant transmissions were considered those with an estimated p-value < 0.00208. Further details on these methods have been reported elsewhere (Angelis et al, 2015; Magiorkinis et al, 2016; Kostaki et al, 2018; Kostaki et al, 2022a).

## 3 Results

### 3.1 Study population characteristics

Our study dataset consisted of 3,093 sequences of HIV-1 subtypes A1 and B which were found previously to belong to large MTCs. We performed a modified phylogeographic analysis on a subset of the study data consisting of 2,385 (subtype A1: 1,229, subtype B: 1,156) sequences with an estimated pairwise genetic distance ≤ 0.015 substitutions/site. The characteristics of PLHIV whose sequences analysed in the context of the current study are presented in Table 1. In brief, most of these people were treatment-naïve (subtype A: 90.9%, subtype B: 86.3%), males (A: 87.3%, B: 91.4%), men who have sex with men (MSM) (A: 67.0%, B: 72.4%), of Greek origin (A: 79.0%, B: 82.4%).

**Table 1:** Characteristics of people living with HIV (PLHIV) infected with HIV-1 subtypes A1 and B whose sequences were included in the modified phylogeographic analysis (n=2,385).

Characteristic	Subtype A1 (n=1,229)	Subtype B (n=1,156)
Antiretroviral therapy (ART) (n, %)		
Untreated	1,117 (90.9)	997 (86.3)
Treated¹	96 (7.8)	88 (7.6)
Unknown status	16 (1.3)	71 (6.1)
Age (median, IQR²)³	34.8 (28.6, 44.2)	32.9 (27.8, 39.6)
Sex (n, %)		
Male	1,073 (87.3)	1,057 (91.4)
Female	93 (7.6)	60 (5.2)
Unknown	63 (5.1)	39 (3.4)
Origin (n, %)		
Greek	971 (79.0)	952 (82.4)
Non-Greek	92 (7.5)	77 (6.6)
Unknown	166 (13.5)	127 (11.0)
Mode of HIV acquisition (n, %)		
Heterosexuals	199 (16.2)	107 (9.3)
PWID⁴	90 (7.3)	122 (10.5)
MSM⁵	824 (67.0)	837 (72.4)
Other	6 (0.5)	9 (0.8)
Unknown	110 (9.0)	81 (7.0)

¹ People living with HIV on ART at genotypic resistance testing

² IQR, interquartile range

³ Age estimation was based on 1,149 and 1,101 available observations for subtypes A1 and B, respectively

⁴ PWID, people who inject drugs

⁵ MSM, men who have sex with men

### 3.2 Analysis based on the definition of late presentation according to the estimated time between HIV acquisition and diagnosis

The total number of sequences assigned to diagnosis-status categories based on the estimated time from HIV infection to diagnosis and after all the exclusions made (described in the methods section), was 1,076 for subtype A1 and 974 for subtype B. For subtype A1, we found that 323 (30%), 325 (30.2%), and 317 (29.5%) treatment-naïve PLHIV were classified as people with recent, intermediate, and late diagnosis, respectively. Furthermore, 111 (10.3%) PLHIV were classified as treated or as having an unknown treatment status. For subtype B, we found that 275 (28.2%), 276 (28.3%), and 273 (28%) treatment-naïve PLHIV were classified as people with recent, intermediate, and late diagnosis, respectively, while 150 (15.5%) PLHIV were on ART at genotypic resistance testing or had an unknown treatment status.

For subtype A1, analysis based on the estimated time from HIV acquisition to diagnosis revealed distinct patterns of transmission between the different groups. Among PLHIV with recent diagnosis, a considerable proportion of infections originated from individuals diagnosed at a late (mean number of infections: 35.7, 11%) or intermediate (mean number of infections: 32.8, 10.2%) stage of diagnosis (Table 2, Fig. 1A). In contrast, individuals diagnosed late acquired infections at lower proportions from those with recent diagnosis (18.5 infections (mean number), 5.8%) and from those with intermediate diagnosis (21.3 infections (mean number), 6.7%) (Table 2, Fig. 1A). For PLHIV with intermediate diagnosis, the mean number of infections traced to those with late diagnosis was higher (48.3, 14.9%) – almost double compared to infections from recently diagnosed individuals (24.7, 7.6%) (Table 2, Fig. 1A). Similarly for subtype B, a considerable proportion of infections among PLHIV with recent (mean number of infections: 33.7, 12.2%) and intermediate (mean number of infections: 36.3, 13.1%) diagnosis was acquired from PLHIV with late diagnosis (Table 2, Fig. 1A). Recently diagnosed PLHIV contributed minimally to onward transmission, providing only for the 6.6% and 4.1% of infections among PLHIV with intermediate and late diagnosis, respectively (Table 2, Fig. 1A). Intermediate presenters contributed at moderate proportions, and specifically 11.5% of the infections among the recent and 9.3% among the late presenters (Table 2, Fig. 1A).

**Table 2:** Mean number and proportion of transmissions between people living with HIV (PLHIV) with different diagnosis-status categories for HIV-1 subtypes A1 and B. PLHIV were categorized into different groups according to the estimated time interval between HIV acquisition and diagnosis dates. Rows indicate the diagnosis-status category from which transmissions originated (“exporting from”), whereas columns indicate the diagnosis-status category to which they were directed (“importing to”).

Subtype A1				
Importing to	Recent	Intermediate	Late	Treated or Unknown
Exporting from				
Recent	-	24.7 (7.6)	18.5 (5.8)	7.0 (6.3)
Intermediate	32.8 (10.2)	-	21.3 (6.7)	5.8 (5.3)
Late	35.7 (11.0)*	48.3 (14.9)*	-	31.3 (28.2)*
Treated or Unknown	4.9 (1.5)*	2.8 (0.9)*	3.0 (1.0)*	-

Subtype B				
Importing to	Recent	Intermediate	Late	Treated or Unknown
Exporting from				
Recent	-	18.3 (6.6)	11.1 (4.1)	7.8 (5.2)
Intermediate	31.6 (11.5)*	-	25.3 (9.3)	12.5 (8.3)
Late	33.7 (12.2)*	36.3 (13.1)*	-	31.2 (20.8)*
Treated or Unknown	5.0 (1.8)	7.5 (2.7)*	7.5 (2.7)*	-

*Statistically significant events between diagnosis-status categories (p-value < 0.00208)

**Fig. 1:**
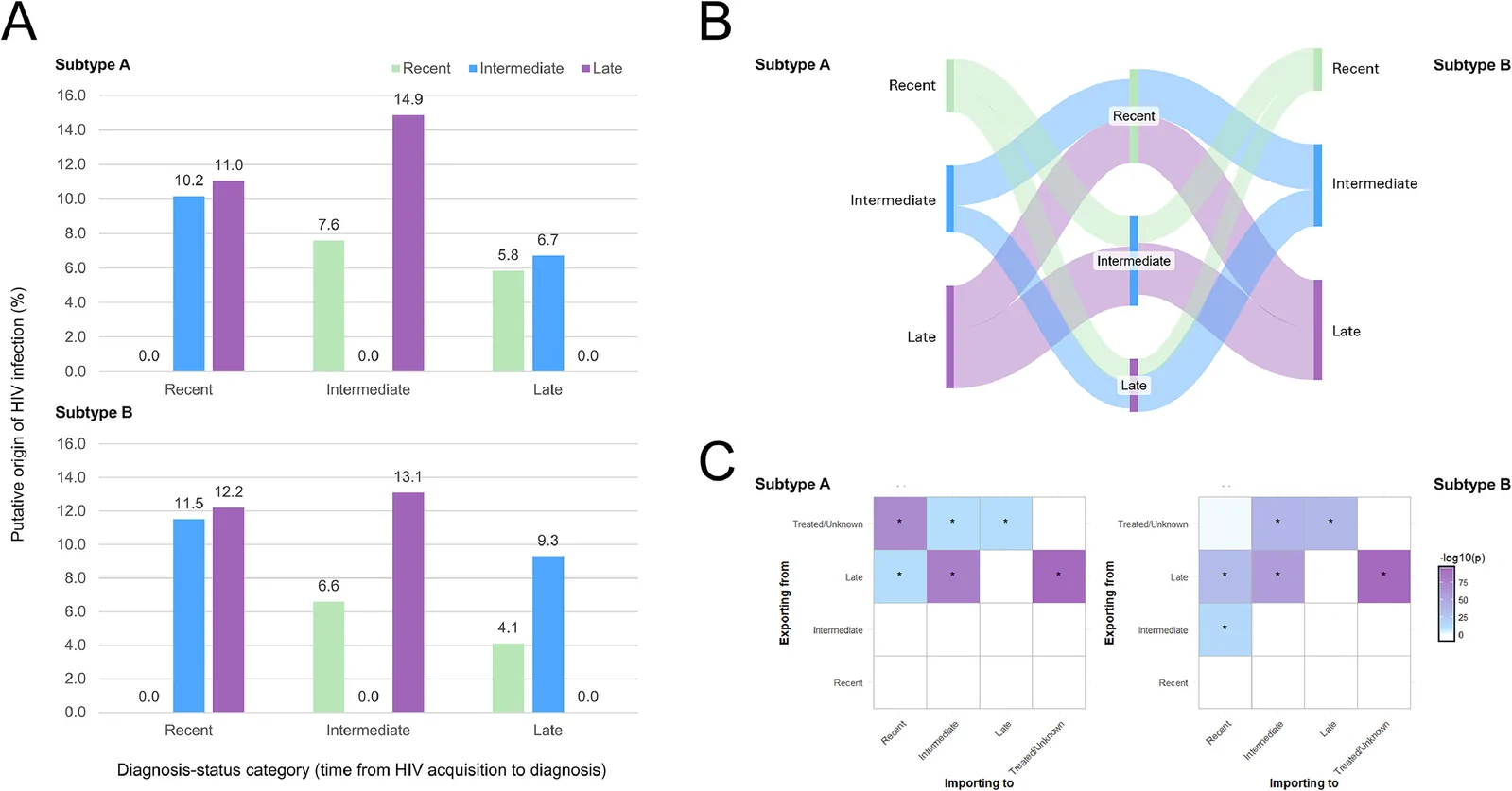
HIV-1 transmissions between people living with HIV (PLHIV) with different diagnosis-status categories for HIV-1 subtypes A1 and B, estimated by a modified phylogeographic analysis performed separately for each subtype. PLHIV were categorized into different groups (recent, intermediate, late, treated or unknown) according to the estimated time interval between HIV acquisition and diagnosis dates. A. Bar plot showing the percentage contribution of PLHIV to onward HIV-1 transmission. B. Sankey diagram depicting the flow of HIV-1 transmissions among PLHIV of different groups, with the flow widths being proportional to the contribution of each group to onward transmission. C. Heatmap illustrating the distribution of HIV-1 transmission events between groups of PLHIV where the color intensity reflects the statistical significance, with stronger color indicating lower p-values (i.e., higher statistical significance). Asterisks denote statistically significant events (i.e., p-value < 0.00208).

The Sankey diagram presented in Fig. 1B, provides a comprehensive visualization of how timing of diagnosis shapes HIV-1 transmission patterns within the study population for both subtypes. Flows represent inferred transmission links, with their width proportional to the contribution of each group to onward transmission. The diagram highlights that individuals diagnosed at later stages contribute a substantial share of onward transmissions. In contrast, recently diagnosed individuals account for a smaller proportion of transmissions.

Statistical phylogeography confirmed that, for both subtypes, PLHIV with late diagnosis provided a significant source of cross-group HIV transmission (Table 2, Figs. 1C, 2A). Specifically, for subtype A1 the number of cross-group transmissions from the late presenters to recently and intermediately diagnosed cases, was significantly higher than those expected by chance only (Table 2, Fig. 1C). For subtype B, late presenters were also identified as a significant source of inter-group transmission (Table 2, Fig. 1C). Additionally, for subtype B, PLHIV with intermediate diagnosis were also found to act as a source of viral transmission to recently diagnosed individuals (Table 2, Fig. 1C). Regarding PLHIV on ART at genotypic resistance testing or with an unknown treatment status, they were also identified as significant sources of transmission (Table 2, Fig. 1C). For subtype A1, this group was a significant source to all groups, while for subtype B, they contributed significantly to intermediate and late presenters (Table 2, Fig. 1C).

**Fig. 2:**
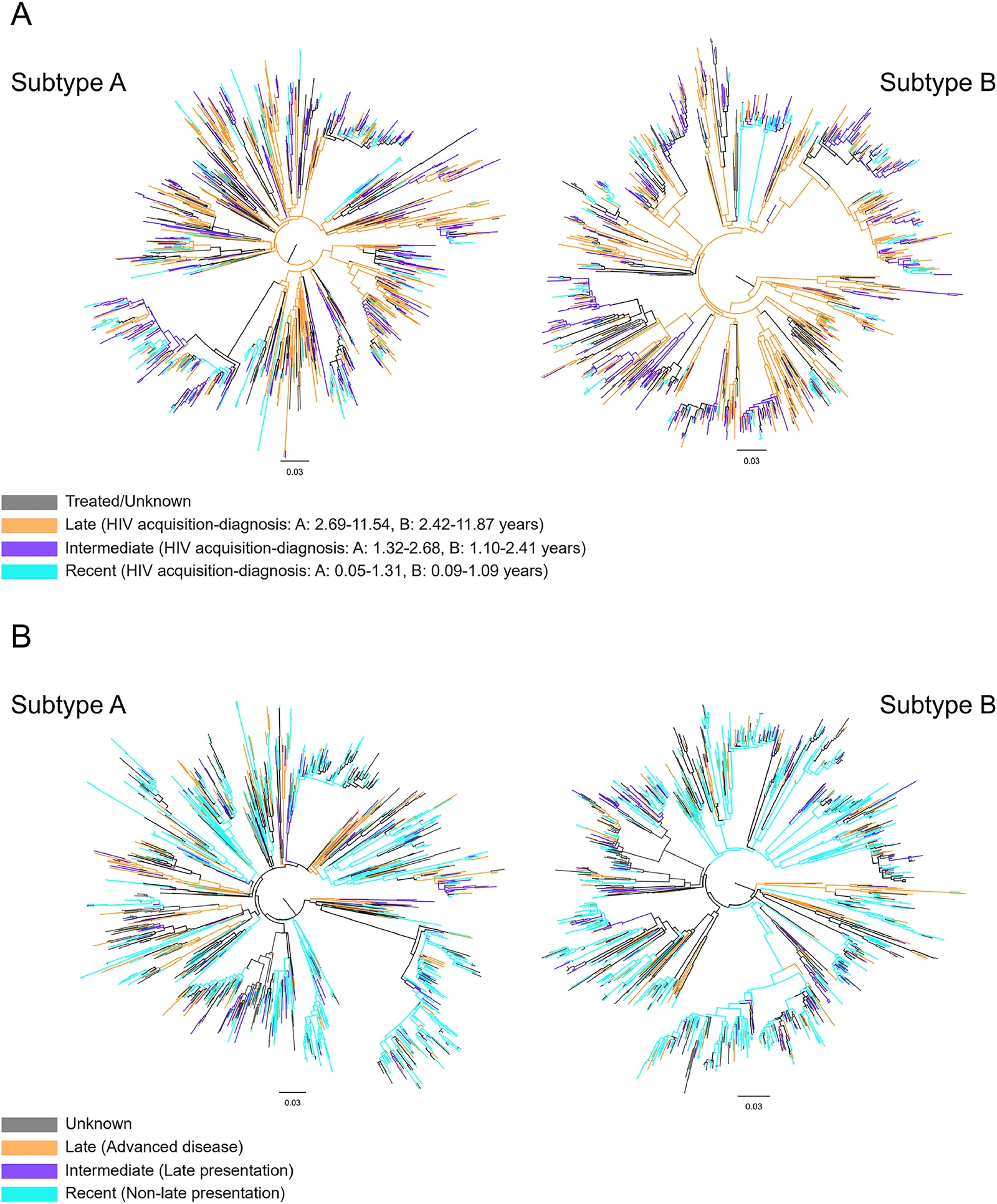
Unrooted maximum likelihood phylogenetic trees estimated by RAxML v8.2.10 using HIV-1 sequences of subtypes A1 and B that were found within large molecular transmission clusters with an estimated pairwise genetic distance ≤ 0.015 substitutions/site and did not fulfil the predefined exclusion criteria. Diagnosis-status categories were assigned as character states to the corresponding sequences (tips) of the phylogenetic trees. Sequences from people living with HIV (PLHIV) with an unknown status of treatment or presentation or on antiretroviral therapy are marked in grey, whereas sequences from PLHIV with available diagnosis-status information are marked in different colors according to the diagnosis status based on the A. estimated time from HIV acquisition to diagnosis and B. CD4 cell count or presence of an AIDS-defining condition at diagnosis. Specifically, light blue, purple, and orange indicate people with recent (non-late presentation), intermediate (late presentation), and late (advanced disease) diagnosis, respectively.

### 3.3 Analysis based on the definition of late presentation according to the CD4 cell count or AIDS-defining condition at diagnosis

The total number of sequences assigned to diagnosis-status categories based on the traditional definition of late presentation according to the CD4 cell count or AIDS-defining condition at diagnosis, and after the exclusion of sequences found within PWID MTCs, was 1,150 for subtype A1 and 1,044 for subtype B. For subtype A1, 230 (20%), 168 (14.6%), and 484 (42.1%) PLHIV were classified as people with advanced disease, late and non-late presentation, respectively. Furthermore, for 268 (23.3%) PLHIV there were no available data for the CD4 cell count or AIDS-defining condition at diagnosis, and as a result they were classified as people with an unknown presentation status. For subtype B, 179 (17.1%), 151 (14.5%) and 442 (42.3%) PLHIV were classified as people with advanced disease, late, and non-late presentation, respectively, while 272 (26.1%) PLHIV had an unknown presentation status. To be consistent with the analysis in the previous section, from this point onward, we will refer to PLHIV with non-late presentation, late presentation and advanced disease as people with recent, intermediate, and late diagnosis, respectively. These labels are used only to facilitate comparison between the two approaches.

In the context of the modified phylogeographic analysis, at the first step, we estimated the number of putative inter-group transmissions. However, since the number of PLHIV in each group differs greatly for both subtypes, direct comparisons of the mean numbers or proportions of intra-group transmissions are not meaningful. For example, for subtype A1, PLHIV with recent diagnosis comprise 42.1% of the sample compared to 20% and 14.6% for those with late and intermediate presentation, respectively. This larger sample size results in a higher number of transmissions originating from this group, making the mean number of viral infections not directly comparable. Therefore, we can focus only on the results of the statistical phylogeographic analysis, which takes into account differences in sample size across the diverse groups.

Statistical phylogeography confirmed that for both subtypes, PLHIV with late diagnosis provided a significant source of cross-group HIV transmission (Figs. 2B and 3B). For subtype A1, statistical phylogeography revealed that PLHIV with late diagnosis were a significant source of transmission to all groups (Fig. 3B). Similarly, PLHIV with intermediate diagnosis contributed significantly to transmissions to all groups (Fig. 3B). In contrast, although being the largest group, PLHIV with recent diagnosis were not identified as a significant source of transmission to any other group (Fig. 3B). A similar pattern was observed for subtype B where those with late diagnosis were a significant source of transmission to all groups, PLHIV with intermediate diagnosis contributed significantly to transmission to recently diagnosed individuals and people with an unknown presentation status, while PLHIV with recent diagnosis were not identified as a significant source of transmission to any other group (Fig. 3B). Moreover, for both subtypes, PLHIV with an unknown presentation status were found to act as sources of transmission only to recently diagnosed individuals (Fig. 3B).

**Fig. 3:**
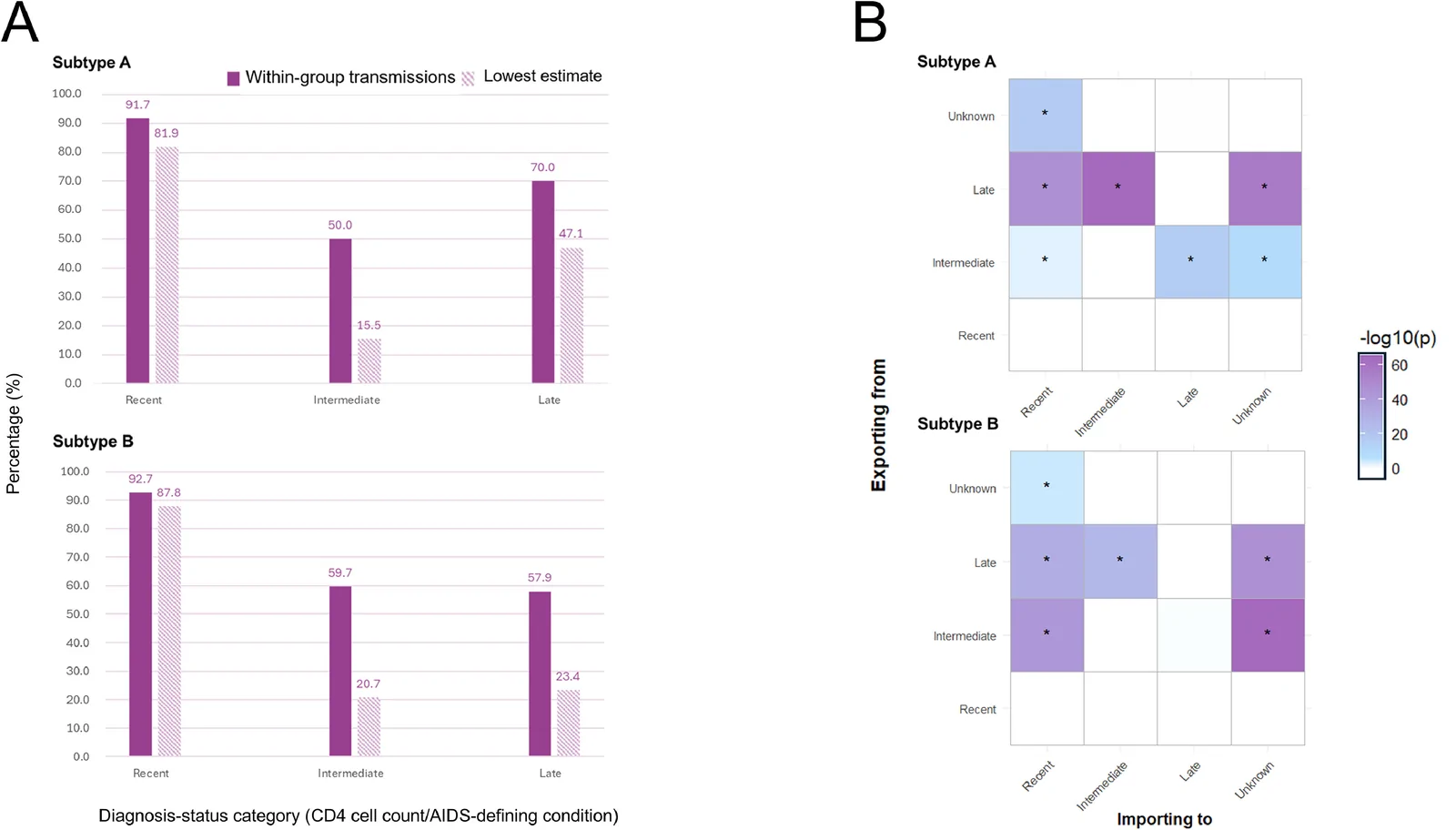
HIV-1 transmissions between people living with HIV (PLHIV) with different diagnosis-status categories for HIV-1 subtypes A1 and B, estimated by a modified phylogeographic analysis performed separately for each subtype. PLHIV were categorized into different groups [recent (non-late presentation), intermediate (late presentation), late (advanced disease), unknown] according to the CD4 cell count or presence of an AIDS-defining condition at diagnosis. A. Bar plot showing the proportion of HIV-1 transmissions within each group, along with the minimum proportion of within-group transmissions estimated taking into account also the ambiguously resolved viral migration events (lowest estimates). B. Heatmap illustrating the distribution of HIV-1 transmission events between groups of PLHIV, where the color intensity reflects the statistical significance, with stronger color indicating lower p-values (i.e., higher statistical significance). Asterisks denote statistically significant events (i.e., p-value < 0.00208).

Based on these findings, we estimated the proportion of HIV-1 transmissions within each group using the CD4-based classification. We used this approach to avoid potential bias from the estimation of infection dates at the t_MRCA_ of the closest node of two sequences. For subtype A1, the proportion of within-group transmissions was 91.7% for recently diagnosed, 50% and 70% for those with intermediate and late diagnosis, respectively, and 65.9% for individuals with an unknown presentation status (Fig. 3A). Considering also the ambiguously resolved viral migration events, the minimum proportions of within-group transmissions were estimated at 81.9% (recent), 15.5% (intermediate), 47.1% (late) and 33% (unknown status) (Fig. 3A). Similarly, for subtype B, the estimated within-group transmission proportions were 92.7% (recent), 59.7% (intermediate), 57.9% (late), and 64.6% (unknown status) (Fig. 3A). The corresponding lowest estimates were 87.8% (recent), 20.7% (intermediate), 23.4% (late), and 33% (unknown status) (Fig. 3A).

## 4 Discussion

Our study provides important findings related to the HIV-1 transmission patterns of the two most prevalent subtypes in Greece using a modified phylogeographic method and two definitions of late presentation – based on the estimated time since infection and the routinely used CD4 cell count/AIDS-defining condition at diagnosis. We showed that late presenters, as defined by both approaches, were consistently identified as the primary source of cross-group transmission across all other groups. This pattern was observed for both HIV-1 subtypes. Moreover, PLHIV with intermediate diagnosis contributed to transmissions to recently diagnosed individuals, for both subtypes using the CD4-based definition, and for subtype B using the time-since-infection definition. In contrast, recently diagnosed cases were not identified as a significant source of transmission to any other group in any of the analyses. Notably, within group transmissions were consistently higher among recently diagnosed individuals for both subtypes.

These findings suggest, first, that the highest proportion of transmissions occurred among PLHIV with recent diagnosis and thus probably during a short period following their infection. However, in addition to this pattern and the within-group transmissions among recently diagnosed individuals, our findings consistently demonstrate that PLHIV with late diagnoses significantly contribute to onward HIV transmission, across both subtypes and under both definitions. Notably, these findings suggest that late presentation, in addition to its well-described negative impact on disease prognosis and immune reconstitution (Müller et al, 2010; Mocroft et al, 2013; Ea Darling et al, 2016; Wijting et al, 2019; Late Presentation Working Groups in EuroSIDA and COHERE, 2020; Rava et al, 2021; Sum et al, 2021), also provides a significant source of HIV transmission to individuals with recent and intermediate diagnoses (Valdiserri, 2007). A key study has shown that the number of HIV late presenters has been correlated with community viral load and that rates among individuals unaware of their HIV status are approximately 3.5 times higher than those diagnosed early (Marks et al, 2006). This is in accordance with a previous study by Chaillon et al. (2016), which showed that delayed HIV diagnosis can contribute to ongoing transmission despite assumptions that these individuals have reduced transmission potential due to lower engagement in high-risk behaviour. Two studies from the United States revealed that around 80% of new HIV infections originate from people who either do not know they are infected or are not receiving regular medical care (Skarbinski et al, 2015; Li et al, 2019). Although the highest transmission rates were observed in those who were acutely infected and unaware, transmission rates remained high even among people with non-acute infection who had never been tested (Li et al, 2019). When we consider that late presenters may go undiagnosed for years, it becomes clear that this prolonged undetected period is itself a major driver of new infections in the community.

Our findings highlight that the transmission potential of late presenters remains high, probably due to a prolonged undiagnosed phase with elevated viral load. In addition, our results showed that individuals with intermediate diagnoses significantly contributed to infections among recently diagnosed PLHIV. This suggests that intermediately diagnosed individuals, those diagnosed later than the early phase but not yet at the late-stage disease, may represent an epidemiologically important group, showing a significant transmission potential. Few studies have explicitly analysed this group, but our findings suggest that interventions focused on this population may be beneficial in reducing HIV incidence.

In contrast, individuals with recent diagnoses – despite being the largest group in both subtypes – did not contribute significantly to onward transmission. This supports the view that early diagnosis and linkage to care may substantially reduce secondary transmission risk, as shown in studies by Cohen et al. (2011) and more recently by ECDC reports highlighting the success of early treatment strategies under the "treatment as prevention" (TasP) framework (ECDC, 2025). Our findings further support the global UNAIDS target (95-95-95) which aimed to significantly reduce HIV transmission by 2030: diagnosing 95% of PLHIV, 95% of those diagnosed with HIV to be linked to care and receive ART, and 95% of people on ART to achieve virological suppression. However, late HIV diagnosis remains a significant global public health challenge. In 2024, it has been increasingly detected reaching 54.2% among newly identified HIV infections in WHO European Region (ECDC/WHO, 2025). The main factors associated with late diagnosis include older age, heterosexual transmission and injection drug use, and migrant status (Vives et al, 2012; Mocroft et al, 2013; Raffetti et al, 2016; Sun et al, 2021; Kostaki et al, 2022b). Notably, our study highlights that late diagnosis contributes also to HIV onward transmission, thus limiting our efforts towards achieving our goal to significantly reduce HIV.

Our findings that the highest proportion of infections occurred among PLHIV with recent diagnosis are consistent with findings from previously reported molecular studies. In particular, similar patterns have been observed in the study by Ratmann et al. (2016), which showed that 43% of the 617 MSM recipients were infected by men on their first year of infection. A strength of their analysis was the integration of phylogenetic analysis with clinical patients’ data, which allowed to infer the timing and direction of potential transmission events. Although a variety of phylogenetic approaches have been used to detect molecular clusters across different geographic areas, the contribution of late presentation to onward transmission has been investigated only in a few studies.

Our study has several strengths, including a dense sampling of HIV-1 sequences, the integration of clinical and molecular data, and the use of a variety of phylogenetic methods, including modified statistical phylogeography and pairwise genetic distances, for tracing the contribution of late presentation on HIV onward transmission. Specifically, we first characterized the presence of molecular transmission clusters and among all sequences within large clusters, we selected only those with pairwise genetic distances likely belonging to transmission pairs. The putative sources of cross-group transmission were then identified by means of modified statistical phylogeography. In this analysis, we excluded the sequences found within MTCs of PWID, as they were characterized by highly similar sequences identified through seek-test-treat (ARISTOTLE programme) and additional targeted public health interventions to address the PWID outbreak in Athens during 2011-2014. However, our study also has limitations such as the incomplete clinical data (CD4 cell count at diagnosis, HIV viral load, HIV infection date) for a subset of individuals, limiting the ability to completely resolve the contribution to onward HIV transmission of those with an unknown presentation status. Moreover, HIV infection dates were approximated using the median t_MRCA_ estimate of the closest paired sequences, suggesting potential bias in their clustering pattern according to their inferred diagnosis status. To address this potential limitation, cross-group transmission patterns were estimated using both definitions of late presentation, while the within-group transmission patterns were estimated using only the CD4-based classification, thus reducing bias. Finally, another limitation of our study is that our population corresponds to PLHIV for whom sequences were available until 2015. Therefore, our findings reflect the situation up to 2015 and should not be interpreted as representative of the subsequent period.

In conclusion, our study provides evidence that PLHIV diagnosed late are a significant source of ongoing HIV transmission, regardless of the HIV-1 subtype or definition of late presentation. These findings highlight the urgent need to enhance testing strategies, promote earlier diagnosis, and ensure rapid linkage to care – not only to improve individual health outcomes but also to interrupt chains of transmission at the population level.

## Ethics declaration

The authors have NOT obtained informed consent from participants or their legal representatives. For this study, fully anonymized nucleotide sequences were retrospectively analyzed; thus, no written informed consent was required.

This study was performed in compliance with relevant laws, regulatory frameworks and guidelines where the research took place. This study was approved by the Ethics Committee of the Medical School of the National and Kapodistrian University of Athens. (Approval No. 1516010195-08/12/2015)

## CRediT authorship contribution statement

**Evangelia Georgia Kostaki:** Writing – review & editing, Writing – original draft, Visualization, Validation, Software, Methodology, Formal analysis, Data curation. **Stefanos Limnaios:** Writing – review & editing, Formal analysis. **Georgios Adamis:** Writing – review & editing, Investigation. **Georgios Xylomenos:** Writing – review & editing, Investigation. **Maria Chini:** Writing – review & editing, Investigation. **Nikos Mangafas:** Writing – review & editing, Investigation. **Marios Lazanas:** Writing – review & editing, Investigation. **Stavros Patrinos:** Writing – review & editing, Data curation. **Simeon Metallidis:** Writing – review & editing, Investigation. **Olga Tsachouridou:** Writing – review & editing, Investigation. **Vasileios Papastamopoulos:** Writing – review & editing, Investigation. **Dimitrios Chatzidimitriou:** Writing – review & editing, Investigation. **Anastasia Antoniadou:** Writing – review & editing, Investigation. **Antonios Papadopoulos:** Writing – review & editing, Investigation. **Konstantinos Protopapas:** Writing – review & editing, Investigation. **Chrysa Tsiara:** Writing – review & editing, Data curation. **Mina Psichogiou:** Writing – review & editing, Investigation. **Dimitrios Basoulis:** Writing – review & editing, Investigation. **Dimitrios Pilalas:** Writing – review & editing, Investigation. **Dimitra Paraskeva:** Writing – review & editing, Investigation. **Georgios Chrysos:** Writing – review & editing, Investigation. **Vasileios Paparizos:** Writing – review & editing, Investigation. **Sofia Kourkounti:** Writing – review & editing, Investigation. **Helen Sambatakou:** Writing – review & editing, Investigation. **Vasileios Bolanos:** Writing – review & editing, Investigation. **Nikolaos V. Sipsas:** Writing – review & editing, Investigation. **Malvina Lada:** Writing – review & editing, Investigation. **Emmanouil Barbounakis:** Writing – review & editing, Investigation. **Evrikleia Kantzilaki:** Writing – review & editing, Investigation. **Periklis Panagopoulos:** Writing – review & editing, Investigation. **Vasilis Petrakis:** Writing – review & editing, Investigation. **Stelios Drimis:** Writing – review & editing, Investigation. **Pagona Lagiou:** Writing – review & editing, Resources. **Angelos Hatzakis:** Writing – review & editing, Resources. **Lemonia Skoura:** Writing – review & editing, Resources. **Gkikas Magiorkinis:** Writing – review & editing, Resources, Methodology. **Dimitrios Paraskevis:** Writing – review & editing, Writing – original draft, Supervision, Resources, Project administration, Methodology, Funding acquisition, Conceptualization.

## Declaration of competing interest

The authors declare that they have no known competing financial interests or personal relationships that could have appeared to influence the work reported in this paper.

## Data Availability

Data will be made available on request.
